# Genetic shifts of Japanese encephalitis virus (JEV) in mosquitoes in the Republic of Korea, 2017–2022

**DOI:** 10.1371/journal.pntd.0013258

**Published:** 2025-07-17

**Authors:** Bo-Ram Yun, Ji-Young Kwon, Byung-Eon Noh, Sehoon Cho, Dongmi Kwak, Hee Il Lee

**Affiliations:** 1 Division of Vectors and Parasitic Diseases, Korea Disease Control and Prevention Agency, Cheongju, Republic of Korea; 2 Kyungpook National University College of Veterinary Medicine, Daegu, Republic of Korea; QIMR: QIMR Berghofer Medical Research Institute, AUSTRALIA

## Abstract

**Background:**

The Japanese encephalitis virus (JEV) is transmitted by mosquitoes and circulates in Asia, the western Pacific, and other parts of the world. It is classified into five genotypes (GI–GV) based on the nucleotide sequence of the envelope (E) gene. Despite extensive surveillance, shifts in genotype distribution and mosquito species remain underreported, particularly in the Republic of Korea.

**Methodology/Principal findings:**

We conducted a nationwide mosquito collection from 2017 to 2022, capturing 1,102,031 mosquitoes from 32 sites nationwide. The predominant species were *Aedes vexans* (34.2%), *Culex pipiens* (17.9%), *Cx. tritaeniorhynchus* (13.4%), *Ae. albopictus* (4.0%), and *Cx. orientalis* (0.6%). JEV was detected in 49 pools, with the majority from *Cx. pipiens* and *Cx. tritaeniorhynchus*. Genetic analyses identified genotypes I, III, and V, with genotype V becoming dominant from 2020 onwards.

**Conclusions/Significance:**

The emergence of genotype V as the dominant strain of the JEV, along with its detection in mosquito species other than the previously known *Cx. tritaeniorhynchus*, highlights the need for ongoing surveillance. These findings underscore the importance of developing vaccines effective against all JEV genotypes to mitigate public health risks.

## Author summary

The Japanese encephalitis virus (JEV) is a mosquito-borne zoonotic virus belonging to the family Flaviviridae, genus *Orthoflavivirus,* which causes severe encephalitis in humans, leading to significant morbidity and mortality. JEV is transmitted primarily through the bites of infected female *Culex tritaeniorhynchus* mosquitoes and can be classified into five different genotypes (GI–GV) that are closely related by envelope gene sequences. Little is known about the evolution, geographical distribution, and epidemiology of these genotypes in the Republic of Korea (ROK) and the potential implications for public health. To address this knowledge gap, we analyzed the spatiotemporal distribution of mosquito populations and investigated the epidemiology of JEV genotypes in the ROK between 2017 and 2022. While *Cx. tritaeniorhynchus* is widely recognized as the primary vector of JEV in Asia, the western Pacific, and other regions, the detection of genotype V (GV) in several mosquito species in this study suggests a potential shift in vector dynamics. Furthermore, these findings indicate the possible re-emergence of GV in Asian countries following its initial isolation in Malaysia in 1952. Consequently, it is imperative to enhance vector surveillance for JEV detection and develop more effective diagnostic tools for genotype V-associated Japanese encephalitis are imperative.

## Introduction

Japanese encephalitis (JE) is a mosquito-borne zoonotic disease that can cause severe encephalitis in humans, with high mortality rates among those who develop encephalitis and long-term neurological sequelae among survivors. [1]. Although the infection is asymptomatic in most adults, children and the elderly are particularly vulnerable, with a fatality rate of around 30% among severe cases [2]. Despite vaccination efforts, JE remains a significant public health issue in Asia, including the Republic of Korea (ROK), where periodic outbreaks have been recorded since 1946. The number of cases has fluctuated over time, but recent data indicate a steady increase, raising concerns about the re-emergence of this disease in the region [3–6].

The Japanese encephalitis virus (JEV), the causative agent of JE, is maintained in a cycle involving *Culex* mosquitoes and animals such as swine and birds. The virus belongs to the family *Flaviviridae*, with a genome encoding structural and non-structural proteins. JEV is classified into five genotypes (GI-GV), primarily based on the nucleotide sequences of the envelope gene. The virus has circulated endemically in Asia and the Pacific region for nearly a century [7]. Transmission to humans occurs primarily through the bites of infected female *Culex tritaeniorhynchus* mosquitoes. In East Asia, however, several mosquito species, including *Cx. tritaeniorhynchus*, *Cx. pipiens* complex, *Cx. vishnui*, *Cx. orientalis,* and *Aedes* spp., are recognized as important JEV vectors, many of which are indigenous to the ROK [1,8–20]. Over the past three decades, genotype III (GIII), previously dominant in many Asian countries, has largely been replaced by genotype I (GI) [21,22]. In the ROK, however, the detection of genotype V (GV), first isolated in Malaysia in 1952, has raised concerns about the efficacy of current GIII-based vaccines against emerging strains [23]. In the ROK, JEV GV was detected in cerebrospinal fluid samples from patients with suspected JE in 2015 and 2018 [6,24]. JEV GV was first detected in mosquitoes in the ROK in 2010, particularly in *Culex bitaeniorhynchus* [25], and has since been found in multiple mosquito species [13,14,24].

Although surveillance of JEV in mosquitoes is ongoing, there is limited data on the distribution of different JEV genotypes in the ROK and the potential implications for public health. This study addresses this gap by reporting the spatiotemporal distribution of mosquito populations and investigating the epidemiology of JEV genotypes in the ROK between 2017 and 2022.

## Materials and methods

### Mosquito sampling

**Fig 1:**
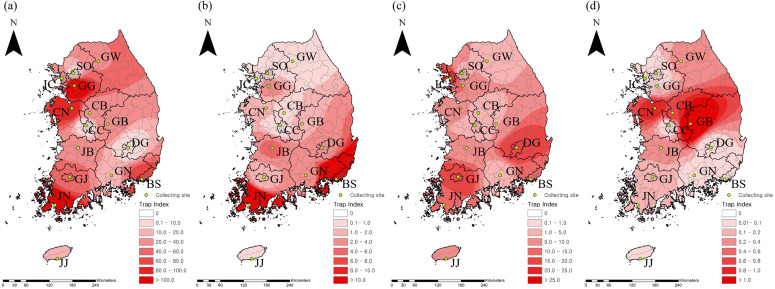
Geographical distribution of major mosquito species transmitting the Japanese encephalitis virus (JEV) in the Republic of Korea from 2017 to 2022 (a) Overall distribution of the three mosquito species. (b) *Cx. tritaeniorhynchus*, (c), *Cx. pipiens* complex, (d) *Cx. orientalis*. The base layer image was sourced from the GADM(https://gadm.org/maps.html). Map color indicates trap index (0 to >100) and collection traps are indicated by green dots. Trap index represents the number of mosquitoes per trap per night. SO, Seoul; IC, Incheon; GG, Gyeonggi; GW, Gangwon; CB, Chungbuk; CN, Chungnam; GB, Gyeongbuk; GN, Gyeongnam; DJ, Daejeon; DG, Daegu; GJ, Gwangju; BS, Busan; JB, Jeonbuk; JN, Jeonnam; JJ, Jeju.

Routine mosquito surveillance was conducted across 15 provinces in the ROK from March to November between 2017 and 2022. Sampling locations included urban areas, rural areas, livestock sheds, and migratory bird habitats in Seoul (SO), Incheon (IC), Gangwon-do (GW), and Gyeonggi-do (GG) in the northern region; Chungcheongbuk-do (CB), Chungcheongnam-do (CN), Gyeongsangbuk-do (GB), Jeollabuk-do (JB), Daejeon (DJ), and Daegu (DG) in the central region; Gyeongsangnam-do (GS), Jeollanam-do (JN), Busan (BS), and Gwangju (GJ) in the southern region; and Jeju (JJ) the southernmost island (Fig 1).

Mosquitoes were collected using four types of traps: dry ice-baited black-light traps (The John W. Hock Co., Gainesville, FL, USA), BG-Sentinel traps (BioGents, Regensburg, Germany), LED traps (UV-LED Blackhole Plus Mosquito trap; Biotrap Co., Ltd., Gyeonggi, ROK) and CO_2_-baited Digital Mosquito Monitoring Systems (DMS; E-TND Co., Ltd., Gyeonggi, ROK). Collections were conducted twice monthly. To assess mosquito densities, the trap index (TI)–the average number of female mosquitoes collected per trap per night–was calculated.

### Morphological identification of mosquito species

Collected mosquitoes were transported to the laboratory in cooler boxes filled with dry ice. After immobilizing them in an ice chamber, the female mosquitoes were morphologically identified using a stereomicroscope (Olympus-SZ61; Tokyo, Japan) and standard taxonomic keys [26]. Identified specimens were stored for subsequent molecular analyses to detect the presence of JEV.

### RNA extraction

Mosquitoes were pooled by species, collection period, and site, with each pool containing a minimum of 1 to a maximum of 30 individuals. Samples were stored at -80 °C until RNA extraction. To homogenize the mosquito pools, glass beads were added, and the pools were processed in RLD buffer (cat. no. IN1003, Invirustech, Gwangju, Republic of Korea) for two 30-second cycles at 7,500 rpm using a Precellys Evolution homogenizer (Bertin Technologies, Bretonneux, France). Viral RNA was extracted using the Clear-S^TM^ Total RNA Extraction Kit (cat. no. IVT7001, Invitrogen, Korea) according to the manufacturer’s instructions. RNA samples were then aliquoted and stored at -80 °C for the reverse-transcription (RT) step.

### Molecular detection of JEV in mosquitoes

The extracted RNA was tested for JEV using reverse-transcription polymerase chain reaction (RT-PCR) to target the non-structural protein 5 (NS5) gene. A Clear-MD flavivirus real-time RT-PCR detection kit (cat. no. IVT-M1001KS, Invirustech, Korea) was used for this purpose. The RT-PCR cycling conditions included an RT step at 45 °C for 10 min for cDNA synthesis, followed by an inactivation step at 95 °C for 10 min. Subsequently, 40 amplification cycles were performed, consisting of 10 s of denaturation at 95 °C, 15 s of annealing at 60 °C, 10 s of extension at 72 °C, and 15 s of signal reading at 80 °C. When the reaction is completed, the samples were cooled to 25 °C to stabilize the reaction products. After real-time RT-PCR, the results were analyzed by setting an appropriate threshold. Samples were considered positive if the cycle of threshold (Ct) value was less than 40 and the melting temperature ranged from 82 °C to 88 °C, as determined by amplification plot and melting curve analysis. The expected amplicon size of the NS5 gene was approximately 250 bp. The minimum infection rate (MIRs) was calculated as:

MIR = number of positive mosquito pools/total number of mosquitoes tested × 1000.

PCR products were confirmed by automated capillary electrophoresis (QIAxcel Advanced System; Qiagen, Hilden, Germany) and sequenced via Sanger sequencing. The sequences were deposited in GenBank under accession numbers PQ121414-PQ121433.

### Phylogenetic analysis

The obtained sequences were proofread and trimmed using MEGA version 11.0. and Finch TV Chromatogram Viewer ver. 1.3.1 (Geospiza, Inc.). Sequence identity was assessed using the BLAST tool in GenBank (https://blast.ncbi.nlm.nih.gov/Blast.cgi). All mosquito pools were tested for JEV, and positive samples were used to infer potential associations between mosquito species and JEV genotypes. Phylogenetic comparisons were made with published sequences of JEV strains from humans and mosquitoes, available in the GenBank database. Phylogenetic trees were constructed using the neighbor-joining method in MEGA 11.0, applying the Jukes and Cantor distance model. Tree robustness was assessed with 1,000 bootstrap replicates.

### Geographical and statistical analyses

Geographical distribution maps were created using the inverse distance weighting technique in the spatial analyst toolset in ArcGIS ver. 10.2 (ArcMap software; Environmental Systems Research Institute Inc. (ESRI), Redlands, CA, USA) to compare the geographical distribution of mosquitoes. For statistical analysis, the MIR was calculated to compare infection rates using the formula: MIR = number of positive mosquito pools/ total number of mosquitoes tested × 1000.

## Results

### Mosquito population surveillance

A total of 1,102,031 mosquitoes, representing two subfamilies, nine genera, and more than 30 species, were collected across 15 regions of the ROK from March to November between 2017 and 2022 (Tables 1 and 2). The most common mosquito species was *Aedes vexans* (34.2%, 376,791/1,102,031, TI = 31.2) followed by *Anopheles* spp. (20.1%, 221,099/1,102,031, TI = 18.3) *Culex pipiens* (17.9%, 196,800/1,102,031, TI = 16.3), *Culex tritaeniorhynchus* (13.4%, 147,789/1,102,031, TI = 12.2). Other species each accounted for less than 5% of the total number of mosquitoes collected (Table 1).

**Table 1:** Geographical distribution of mosquito species collected from the Republic of Korea in 2017–2022.

Region	No. of Collected Mosquitoes															
	*Ae. vex*	*Anopheles* spp.	*Cx. pip*	*Cx. tri*	*Ar. sub*	*Ae. albo*	*Och. dor*	*Och. kor*	*Cx. ori*	*Man. uni*	*Cx. ina*	*Och. tog*	*Cx. vag*	*Cx. bit*	*Ae. lin*	Others
Gangwon	25,699	11,661	6,062	417	7,885	5,598	29	2,374	328	39	–	69	47	67	1	75
Gyeonggi	123,159	40,053	14,578	4,009	1,779	759	23,367	1,213	161	121	237	–	56	256	431	12
Gyeongnam	6,070	6,398	3,952	3,736	1,602	3,920	6	992	123	12	10	70	220	17	1	136
Gyeongbuk	24,501	8,762	7,456	2,675	3,041	3,097	1	655	2,432	5	–	21	25	186	57	38
Gwangju	22,855	24,006	24,881	1,609	931	1,750	6	371	267	209	–	7	–	23	–	50
Daegu	11,651	13,391	28,152	4,124	9,192	7,879	–	436	58	14	–	–	2	281	–	6
Daejeon	459	487	1,313	31	2,086	1,124	–	748	53	–	–	1	3	–	1	31
Busan	11,631	4,328	18,710	63,953	2,536	1,760	96	431	98	1,476	1	1,454	–	120	–	53
Seoul	2,062	167	5,865	11	536	1,625	9	2,173	4	1	27	–	12	2	–	1
Incheon	4,258	847	31,855	311	18	1,190	4,881	293	111	189	1,668	7	72	108	506	41
Jeonnam	39,161	65,990	18,419	57,925	3,200	5,833	1,791	794	163	954	74	118	4	69	4	104
Jeonbuk	5,542	12,320	11,185	6,218	1,303	646	59	423	327	2,743	418	7	54	251	46	221
Jeju	24	2,344	11,290	458	1,912	2,042	44	171	2	6	4	157	2	6	–	3
Chungnam	89,136	26,795	8,276	1,659	3,111	4,682	15	1,079	1,336	12	320	6	1,244	38	1	123
Chungbuk	10,583	3,550	4,806	653	7,857	2,210	2	3,649	1,203	29	–	25	–	30	1	25
Collected (%)	376,791 (34.2)	221,099 (20.1)	196,800 (17.9)	147,789 (13.4)	46,989 (4.3)	44,115 (4.0)	30,306 (2.8)	15,802 (1.4)	6,666 (0.6)	5,810 (0.5)	2,759 (0.3)	1,942 (0.2)	1,741 (0.2)	1,454 (0.1)	1,049 (0.1)	919 (<0.1)
TI^*^	31.2	18.3	16.3	12.2	3.9	3.6	2.5	1.3	0.6	0.5	0.2	0.2	0.1	0.1	0.1	0.1

*Ae. vex, Aedes vexans*; *Cx. pip*, *Culex pipiens* complex; *Cx. tri*, *Cx. tritaeniorhynchus*; *Ar. Sub, Armigeres subalbatus*; *Ae. albo, Aedes albopictus*; *Och. dor, Ochlerotatus dorsalis*; *Och. kor, Ochlerotatus koreicus*; *Cx. ori*, *Culex orientalis*; *Man. uni, Mansonia uniformis*; *Cx. ina, Culex inatomii*; *Och. tog, Ochlerotatus togoi*; *Cx. vag, Culex vagans*; *Cx. bit, Culex bitaeniorhynchus*; *Ae. lin, Aedes lineatopennis*. Mosquitoes for which we only identified the *genus* were recorded in the column indicated “*Genus* spp.” Others <0.1 (0.08%).

* TI, trap index (no. of mosquitoes collected/no. of installed traps/nights.

**Table 2:** Summary of mosquito species, number of mosquitoes, and pools tested in the study collected in the Republic of Korea between 2017 and 2022.

Species	No. of Mosquitoes										
	March	April	May	June	July	August	September	October	November	collected (%)	TI^*^
*Cx. tri*	7	44	169	3,550	46,239	57,005	38,738	1,953	84	147,789 (13.4)	12.2
*Cx. pip*	1,020	3,790	11,678	50,419	53,982	20,987	22,080	19,811	13,033	196,800 (17.9)	16.3
*Cx. ori*	6	80	98	706	2,182	2,295	1,233	54	12	6,666 (0.6)	0.6
*Ae. albo*	6	49	359	2,304	8,342	13,922	15,185	3,579	369	44,115 (4.0)	3.6
*Ae. vex*	1	61	34,555	113,189	95,789	89,690	39,573	3,856	77	376,791 (34.2)	31.2
Others	311	1,189	8,463	62,705	141,410	62,411	39,060	12,958	1,363	329,870 (29.9)	27.1
Total	1,351	5,213	55,322	232,873	347,944	246,310	155,869	42,211	14,938	1,102,031 (100.0)	91.2

*Cx. tri, Cx. tritaeniorhynchus; Cx. pip, Culex pipiens complex; Cx. ori, Culex orientalis; Ae. albo, Aedes albopictus; Ae. vexans, Aedes vexans*; Others, other species.

* TI, trap index (no. of mosquitoes collected/no. of installed traps/nights).

The geographical distribution of major *Culex* species known as vectors of the JEV in the ROK was analyzed [13]. Geographical analysis revealed that *Cx. tritaeniorhynchus* was predominantly found in Busan (43.3%, 63,953/147,789) and Jeonnam (39.2%, 57,925/147,789). *Cx. pipiens* complex was mainly collected in Incheon (16.2%, 31,855/196,800), Daegu (14.3%, 28,152/196,800), and Gwangju (12.6%, 24,881/196,800). *Cx. orientalis* was most prevalent in Gyeongbuk (36.5%, 2,432/6,666), followed by Chungnam (20.0%, 1,336/6,666), and Chungbuk (20.0%, 1,203/6,666). (Table 1 and Fig 1). Seasonal fluctuations were observed among the three *Culex* species. The populations of both *Cx. tritaeniorhynchus* and the *Cx. pipiens* complex increased sharply in June (by approximately 21-fold, 4.3-fold, respectively), while *Cx. orientalis* showed a marked increase in April (by approximately 13.3-fold). The highest number of *Cx. tritaeniorhynchus* was recorded in August, whereas those of both the *Cx. pipiens* complex and *Cx. orientalis* peaked in July. Additionally, the *Cx. pipiens* complex declined after August, while *Cx. tritaeniorhynchus* and *Cx. orientalis* began to decrease after September (Table 2 and S1 Fig). From 2017 to 2022, *Cx. tritaeniorhynchus* was primarily collected in Busan and Jeollanam-do. *Cx. pipiens* complex was mostly collected in Daegu and Jeollanam-do in 2017, whereas it was most abundant in Incheon from 2018 to 2021 and Gwangju in 2022. *Cx. orientalis* was predominantly collected in Gyeongsangbuk-do from 2017 to 2022, except in 2020 (S2 Fig).

### Detection of JEV in mosquitoes

Among the 1,102,031 mosquitoes collected from 15 regions between 2017 and 2022, 49 pools tested positive for JEV, yielding a MIR of 0.07 (49 positive pools/736,243 mosquitoes) (Table 3). Specifically, 18 of 4,096 pools of *Cx. tritaeniorhynchus* (MIR = 0.14; 18 pools/132,336 mosquitoes), 19 of 9,517 pools of *Cx. pipiens* (MIR = 0.11; 19 pools/171,089 mosquitoes), and eight of 1,200 pools of *Cx. orientalis* (MIR = 1.34; 8 pools/5,981 mosquitoes) were JEV-positive. Additionally, two of 3,264 pools of *Aedes albopictus* (MIR = 0.05; 2 pools/40,767 mosquitoes) and two of the 9,861 pools of *Ae. vexans* (MIR = 0.01; two pools/296,087 mosquitoes) tested positive for JEV.

**Table 3:** Summary of the JEV detected from different mosquito species collected in the Republic of Korea between 2017 and 2022.

	2017			2018			2019			2020			2021			2022		
	Collected (%)	Tested (pools)	Positive (MIR*)	Collected (%)	Tested (pools)	Positive (MIR)	Collected (%)	Tested (pools)	Positive (MIR)	Collected (%)	Tested (pools)	Positive (MIR)	Collected (%)	Tested (pools)	Positive (MIR)	Collected (%)	Tested (pools)	Positive (MIR)
*Cx. tri*	78,057 (26.1)	77,995 (1,815)	17 (0.22)	25,634 (13.4)	25,162 (749)	1 (0.04)	9,964 (3.8)	9,964 (516)	–	15,316 (13.8)	15,301 (679)	–	4,908 (4.2)	1,393 (163)	–	13,910 (11.5)	2,521 (174)	–
*Cx. Pip*	33,766 (11.3)	33,109 (1,495)	5 (0.15)	28,390 (14.8)	27,057 (1,276)	4 (0.15)	33,147 (12.6)	32,667 (1,907)	4 (0.12)	30,530 (27.6)	28,986 (1,784)	–	36,649 (31.4)	26,837 (1,674)	4 (0.15)	34,318 (28.4)	22,433 (1,381)	2 (0.09)
*Cx. Ori*	1,041 (0.3)	969 (234)	–	1,030 (0.5)	663 (166)	1 (1.51)	1,617 (0.6)	1,617 (263)	2 (1.24)	1,395 (1.3)	1,386 (241)	3 (2.16)	1,112 (1.0)	1,036 (179)	1 (0.97)	471 (0.4)	310 (117)	1 (3.23)
*Ae. albo*	9,195 (3.1)	8,793 (548)	2 (0.23)	7,696 (4.0)	6,158 (478)	–	8,180 (3.1)	8,180 (657)	–	8,813 (8.0)	8,767 (679)	–	6,265 (5.4)	5,489 (524)	–	3,966 (3.3)	3,380 (378)	–
*Ae. vex*	101,178 (33.9)	99,769 (2,485)	–	73,294 (38.3)	66,055 (1,621)	–	129,619 (49.1)	95,160 (3,623)	2 (0.02)	20,835 (18.8)	16,365 (920)	–	24,128 (20.7)	10,880 (686)	–	27,737 (22.9)	7,858 (526)	–

* MIR (minimum infection rate (no. of positive mosquito pools/total no. of mosquitoes tested * 1000). *Cx. tri*, *Cx. tritaeniorhynchus*; *Cx. pip*, *Cx. pipiens* complex; *Cx. ori*, *Cx. orientalis*; *Ae. albo, Aedes albopictus*

The highest number of JEV-positive pools was detected in 2017 (24 pools), while the lowest numbers were recorded in 2020 and 2022 (3 pools each) (Table 3). The MIR was highest in 2017 (MIR = 0.10, 24 pools/234,755 mosquitoes) and lowest in 2020 (MIR = 0.03, 3 pools/85,903 mosquitoes). Across the 49 JEV-positive pools, *Cx. pipiens* complex had the most detections (19/49), followed by *Cx. tritaeniorhynchus* (18/49), and *Cx. orientalis* (8/49) (Table 4). Notably, *Cx. pipiens* complex tested positive for JEV each year except 2020, while *Cx. tritaeniorhynchus* was only detected as positive in 2017–2018. *Cx. orientalis* has been consistently positive for JEV since 2018.

**Table 4:** Detection and identification of JEV genotypes in the mosquito pools.

		No. of JEV-Positive Pools					
Year	Genotypes	*Culex tritaeniorhynchus*	*Culex pipiens* complex	*Culex orientalis*	*Aedes albopictus*	*Aedes vexans*	Total
2017	**Subtotal**	**17**	**5**		**2**		**24**
	GI	10					10
	GIII	7	4		2		13
	GV		1				1
2018	**Subtotal**	**1**	**4**	**1**			**6**
	GI	1					1
	GV		4	1			5
2019	**Subtotal**		**4**	**2**		**2**	**8**
	GIII					1	1
	GV		4	2		1	7
2020	**Subtotal**			**3**			**3**
	GV			3			3
2021	**Subtotal**		**4**	**1**			**5**
	GV		4	1			5
2022	**Subtotal**		**2**	**1**			**3**
	GV		2	1			3
	**Total**	**18**	**19**	**8**	**2**	**2**	**49**

### Shifting genotypes of JEV in ROK

**Fig 2:**
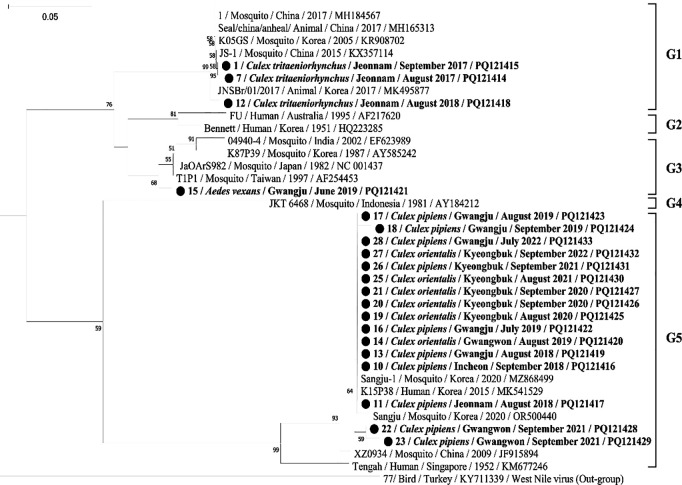
Phylogenetic relationship of the JEV strains (highlighted) using NS5 gene nucleotide sequence homologies with selected JEV reference strains. The tree was generated by the neighbor-joining method. West Nile virus (GenBank accession No. KY711339), a closely related member of the *Orthoflavivirus* genus was used as an outgroup. Bootstrap confidence limits for 1,000 replicates are indicated above each branch. Numbers at nodes indicate bootstrap percentage (over 50%) based on 1000 resampled datasets. The scale bar shows the number of nucleotide substitutions per site.

Of the JEV-positive samples, only those with successfully obtained sequences were included in the phylogenetic analysis. To avoid redundancy, representative sequences were selected when multiple samples originated from the same mosquito species and genotype. In total, 20 sequences were used for phylogenetic tree construction using short sequences of the NS5 gene. The corresponding GenBank accession numbers for these 20 sequences are PQ121414–PQ121433. The evolutionary relationships between these JEV strains and related strains were inferred by constructing a neighbor-joining tree based on the NS5 gene (Fig 2). The West Nile virus was used as the outgroup. The ROK JEV are strains grouped into three distinct clusters: GI, GIII, and GV. Specifically, 11 GI and 7 GIII detections were made in *Cx. tritaeniorhynchus*, 4 GIII, and 15 GV were made in *Cx. pipiens* complex, 8 GV were made in *Cx. orientalis*, and 2 GIII were made in *Ae. Albopictus,* and GIII was detected once in *Ae. vexans*.

In 2017, of the 24 positive pools, 13 were GIII, 10 were GI, and 1 was GV. In 2018, GI and GV were detected in 1 and 5 pools, respectively. Ian 2019, GIII and GV were found in 1 and 7 pools, respectively. From 2020 to 2022, only GV were detected, with three pools testing positive in 2020 and five pools in 2021 and 2022 (Table 4). These results indicate that the genotype V of JEV has become dominant in the ROK. In addition of traditional vector of *Cx. tritaeniorhynchus*, other *Culex* spp. mosquitoes and even *Aedes* spp. mosquitoes can also serve as vectors for the JEV.

## Discussion

Mosquito surveillance is a critical tool for understanding species distribution, infection rates, circulating virus genotypes, and for informing disease control strategies. This study provides valuable insights into the density and diversity of Japanese encephalitis virus (JEV)-infected mosquito species in the ROK from 2017 to 2022.

The major mosquito species associated with mosquito-borne diseases include the *Cx. pipiens* complex (17.9%), *Cx. tritaeniorhynchus* (13.4%), *Ae. albopictus* (4.0%) and *Cx. orientalis* (0.6%). Notably, in 2017, *Cx. tritaeniorhynchus* was the most prevalent species, but from 2018 onward, *Cx. pipiens* complex dominated (S1 Fig). The *Cx. pipiens* complex is known to transmit not only JEV but also the West Nile virus and Zika virus [12,27–31]. We observed that *Cx. tritaeniorhynchus* was primarily localized to the southern regions, while *Cx. pipiens* complex was found across the entire country and *Cx. orientalis* was concentrated in central areas. Furthermore, *Cx. tritaeniorhynchus* and *Cx. pipiens* complex were collected mostly from May to August each year, while *Cx. orientalis* peaked in July and declined by August (Table 2 and S1 Fig). Our study confirmed that *Cx. orientalis*, despite having a shorter active period, exhibited a higher JEV-positive rate (TI = 3.0, MIR = 1.34) compared to other vectors. This suggests that the role of *Cx. orientalis* in JEV transmission warrants further investigation.

While *Cx. tritaeniorhynchus* remains the primary JEV vector in Korea and other Asian countries [11,32,33], other species, including *Cx. bitaeniorhyncus*, *Cx. pipiens* complex, *Aedes* spp., and *Anopheles* spp. have been identified as secondary vectors, contributing to the increased risk of JEV transmission in humans [1,12,13,24,34]. These secondary vectors may play a crucial role in JEV maintenance, particularly in urban and peri-urban environments where *Cx. tritaeniorhynchus* populations are relatively less dominant. In our study, we detected JEV in 49 pools, including *Cx. tritaeniorhynchus* (MIR = 0.14), *Cx. pipiens* complex (0.11), *Cx. orientalis* (1.34), and *Aedes* spp. (0.03). Notably, the MIR for *Cx. orientalis* was significantly higher than the primary vector, suggesting their role in JEV transmission may be underestimated. This highlights the potential role of diverse mosquito species in JEV transmission in the ROK, corroborating previous research that reported JEV isolation or detection in species such as *Cx. pipiens*, *Ae. vexans*, and *Cx. bitaeniorhynchus* in various East Asian regions [1,12,25,42]. Given the adaptability of these secondary vectors to varying ecological conditions, their involvement in JEV transmission may previously have been underestimated. Furthermore, recent changes in climate and land use patterns could be facilitating the expansion of these vectors, potentially increasing the risk of JEV outbreaks. These findings emphasize the need for comprehensive vector surveillance and targeted control strategies to mitigate the risk of JEV transmission in diverse ecological settings. During the study period, JEV circulation was not limited to the ROK but was also reported continuously in neighboring East and Southeast Asian countries including Japan, Thailand, the Philippines, and Indonesia between 2016 and 2018, supporting the notion that the virus remains regionally endemic [43].

Phylogenetic analysis of JEV NS5 gene sequences isolated from infected mosquitoes revealed a shift in the predominant genotype, from GIII to GI, and most recently, to genotype V (Table 4). Historically, JEV genotype III was most commonly detected in human cases until the 1990s, but over the past three decades, the dominant genotype has shifted to GI in regions such as Korea, China, and Japan [21,22]. More recently, JEV GV has emerged, particularly in human cases from China and South Korea [6,35]. In South Korea, JEV GV was first detected in human cerebrospinal fluid samples in 2015 and 2018 [6,24]. Since the initial detection of JEV GV in *Culex bitaeniorhynchus* mosquitoes in 2010, both GI and GV have been consistently identified in mosquito species in Korea [8,12,14,36]. Our findings align with this observed shift in genotype distribution during mosquito surveillance.

This study’s strength lies in its comprehensive surveillance across multiple regions and years, providing a robust dataset for understanding JEV dynamics. We acknowledge the potential for sampling bias in our study, which may have been influenced by local environmental factors, trap placement, and temporal variations in mosquito abundance. While we endeavored to minimize these biases by conducting surveillance across multiple regions and years using standardized trapping protocols, certain areas or time periods may have been underrepresented. This limitation should be considered when interpreting the findings, as it may affect the generalizability of our results.

In this study, we found that *Cx. pipiens* complex and *Cx. orientalis* primarily carried JEV GV, while *Cx. tritaeniorhynchus* predominantly carried GI and GIII (Table 4). Notably, all the JEV strains isolated from *Cx. pipiens* mosquitoes in 2012 and from *Cx. orientalis* mosquitoes in 2020 were GV [14,36]. However, *Cx. tritaeniorhynchus* is recognized as the main JEV vector, the emergence of GV in *Cx. pipiens* complex and *Cx. orientalis* mosquitoes highlights a possible shift in vector dynamics. However, this does not provide direct evidence of their competency as JEV vectors. Studies have shown that European populations of *Cx. pipiens* are capable of transmitting JEV GIII and GV in laboratory settings [27]. The observed genotype shift highlights the need for continued monitoring of JEV genotypes and mosquito vectors. Over the past decade, the predominant JEV genotypes in Asia have shifted from GI and GIII to a more diverse set of genotypes. Recent reports suggest that GV may be re-emerging in parts of Asia, raising questions about its broader public health implications. GV strains have been detected in mosquitoes and/or human cases in China [8], South Korea [12], and more recently in regions such as Tibet, China [44]. Although genotype I (GI) remains the predominant circulating genotype in most Asian countries, the sporadic detection and expanding geographic distribution of GV highlight the need for enhanced surveillance across the region. The re-emergence of GV may be attributed to factors such as increased vector movement, environmental changes, and evolving susceptibility of mosquito species, underscoring the importance of adapting public health strategies to monitor and control potential outbreaks.

The JEV vaccines currently used in South Korea are based on GIII. Although GV has emerged and is widely circulating across Asia, studies evaluating the protective efficacy of existing vaccines against GV strain remain limited [13]. Additionally, the short duration of viremia in JE patients makes it difficult to determine the infecting genotype, and only three cases of JEV GV have been reported in South Korea over the past decade [24,37,38]. If GV continues to expand across Asia, its public health impact will largely depend on vaccine efficacy and vector control measures. Current JEV vaccines, primarily developed based on GI and GIII strains, may offer some cross-protection against GV; however, further studies are needed to assess the extent and durability of this immunity [39–41]. Developing vaccines that provide broad protection, including coverage against GV, is essential to mitigating this public health threat. Countries with strong JEV vaccination programs, such as Japan and Taiwan, may be better prepared for potential outbreaks, whereas regions with lower vaccine coverage could face a heightened risk of transmission.

Continued surveillance of vector mosquito species is essential for tracking emerging JEV genotypes and mitigating the risks to human health. The data from this study can be used to identify key vectors contributing to JEV transmission in ROK. Future research should focus on validating the vector competence of various mosquito species for new JEV genotypes and developing vaccines effective against emerging strains. Additionally, expanding surveillance to include more regions and mosquito species will provide a more comprehensive understanding of JEV epidemiology.

## Supporting information

S1 Fig Monthly and annual distribution of *Cx. tritaeniorhynchus*, *Cx. pipiens* complex, and *Cx. orientalis* in ROK from 2017 to 2022. (a) Monthly distribution (b) Annual distribution trends.
(DOCX)

S2 Fig Annual variation in the population of three *Culex* species from 2017 to 2022. (a) *Cx. tritaeniorhynchus*, (b) *Cx. pipiens* complex, and (c) *Cx. orientalis*.
(DOCX)
