# Supplementary material for: Genetic shifts of Japanese encephalitis virus (JEV) in mosquitoes in the Republic of Korea, 2017–2022
Source: PLoS Negl Trop Dis. 2025 Jul 17;19(7):e0013258. doi: 10.1371/journal.pntd.0013258 (PMC12289085; doi:10.1371/journal.pntd.0013258)
Supplement: S1 Fig — (a) Monthly distribution (b) Annual distribution trends. (DOCX) [file pntd.0013258.s001.docx]

**Supporting Information**


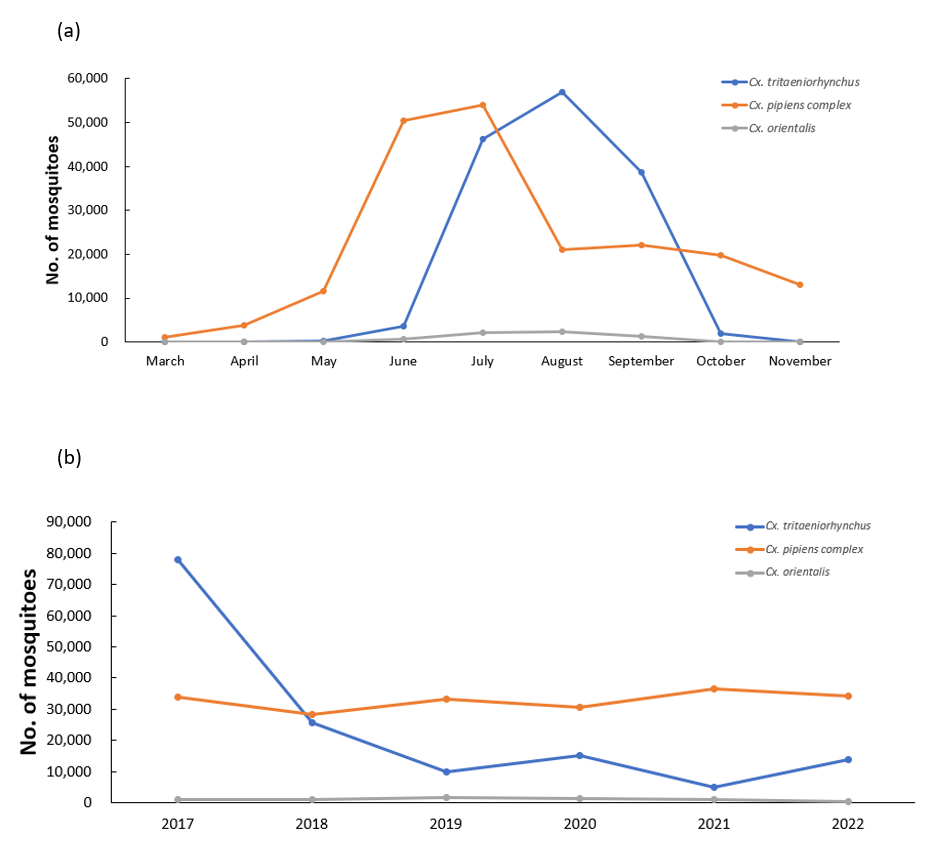


**Figure S1. Monthly and annual distribution of *Cx. tritaeniorhynchus*, *Cx. pipiens* complex, and *Cx. orientalis* in ROK from 2017 to 2022. (a) Monthly distribution (b) Annual distribution trends**
