# Supplementary material for: Genetic shifts of Japanese encephalitis virus (JEV) in mosquitoes in the Republic of Korea, 2017–2022
Source: PLoS Negl Trop Dis. 2025 Jul 17;19(7):e0013258. doi: 10.1371/journal.pntd.0013258 (PMC12289085; doi:10.1371/journal.pntd.0013258)
Supplement: S2 Fig — (a) Cx. tritaeniorhynchus, (b) Cx. pipiens complex, and (c) Cx. orientalis. (DOCX) [file pntd.0013258.s002.docx]

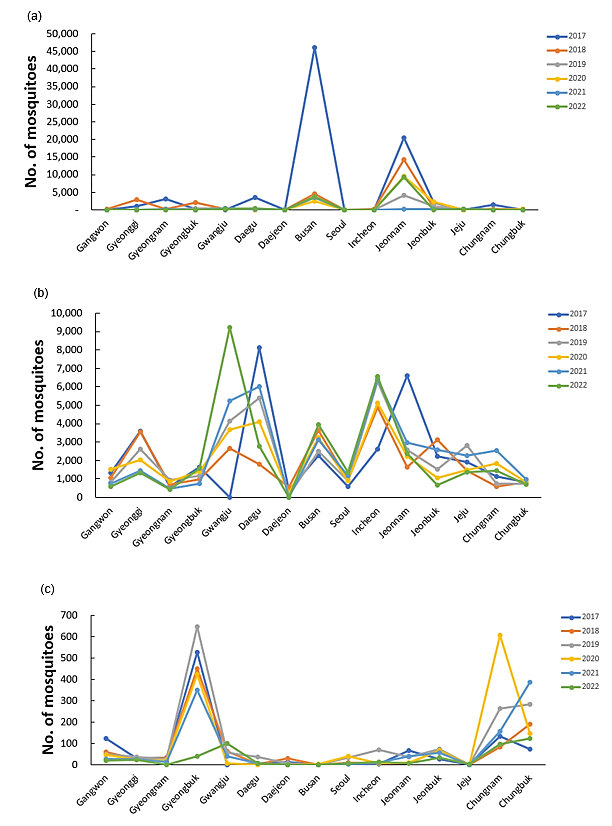


**Figure S2. Annual variation in the population of three *Culex* species from 2017 to 2022. (a) *Cx. tritaeniorhynchus*, (b) *Cx. pipiens* complex, and (c) *Cx. orientalis*.**
